# Non-normal Distributions Commonly Used in Health, Education, and Social Sciences: A Systematic Review

**DOI:** 10.3389/fpsyg.2017.01602

**Published:** 2017-09-14

**Authors:** Roser Bono, María J. Blanca, Jaume Arnau, Juana Gómez-Benito

**Affiliations:** ^1^Social Psychology and Quantitative Psychology, Faculty of Psychology, University of Barcelona Barcelona, Spain; ^2^Institute of Neurosciences, University of Barcelona Barcelona, Spain; ^3^Psychobiology and Behavioral Sciences Methodology, Faculty of Psychology, University of Málaga Málaga, Spain

**Keywords:** non-normal distributions, gamma distribution, negative binomial distribution, multinomial distribution, binomial distribution, lognormal distribution, exponential distribution, systematic review

## Abstract

Statistical analysis is crucial for research and the choice of analytical technique should take into account the specific distribution of data. Although the data obtained from health, educational, and social sciences research are often not normally distributed, there are very few studies detailing which distributions are most likely to represent data in these disciplines. The aim of this systematic review was to determine the frequency of appearance of the most common non-normal distributions in the health, educational, and social sciences. The search was carried out in the Web of Science database, from which we retrieved the abstracts of papers published between 2010 and 2015. The selection was made on the basis of the title and the abstract, and was performed independently by two reviewers. The inter-rater reliability for article selection was high (Cohen’s kappa = 0.84), and agreement regarding the type of distribution reached 96.5%. A total of 262 abstracts were included in the final review. The distribution of the response variable was reported in 231 of these abstracts, while in the remaining 31 it was merely stated that the distribution was non-normal. In terms of their frequency of appearance, the most-common non-normal distributions can be ranked in descending order as follows: gamma, negative binomial, multinomial, binomial, lognormal, and exponential. In addition to identifying the distributions most commonly used in empirical studies these results will help researchers to decide which distributions should be included in simulation studies examining statistical procedures.

## Introduction

The data obtained in many fields of health, education, and the social sciences yield values of skewness and kurtosis that clearly deviate from those of the normal distribution (Micceri, 1989; Lei and Lomax, 2005; Bauer and Sterba, 2011; Blanca et al., 2013). In his imaginatively titled article ‘The Unicorn, The Normal Curve, and Other Improbable Creatures,’ Micceri (1989) concluded that real data commonly follow non-normal distributions. His analysis of the distributional characteristics of over 440 large-sample achievement and psychometric measures revealed several classes of deviation from the normal distribution, with the highest percentage corresponding to extreme deviation. In a more recent study, Blanca et al. (2013) analyzed the shape of 693 distributions from real psychological data by examining the values of the third and fourth central moments as a measurement of skewness and kurtosis in small samples. They found that most distributions were non-normal; considering skewness and kurtosis jointly the results indicated that only 5.5% of the distributions were close to expected values under normality. Overall, 74.4% of distributions presented either slight or moderate deviation, while 20% showed more extreme deviation.

Variables with skewed distributions are also commonly used in a variety of psychological and social research. Arnau et al. (2014) listed some of these variables: reaction times or response latency in cognitive studies (Ulrich and Miller, 1993; Van der Linden, 2006; Shang-Wen and Ming-Hua, 2010), survival data from clinical trials (Qazi et al., 2007), clinical assessment indexes in drug abuse research (Deluchi and Bostrom, 2004), physical and verbal violence in couples (Szinovacz and Egley, 1995; Soler et al., 2000), divorced parents’ satisfaction with co-parenting relationships in family studies (McKenry et al., 1999), and labor income (Diaz-Serrano, 2005) or health care costs (Zhou et al., 2009) in sociological studies. More recent examples involving non-normal data include neuropsychological data (Donnell et al., 2011; Oosthuizen and Phipps, 2012), data about paranoid ideation (Bebbington et al., 2013), fatigue symptoms of breast cancer patients (Ho et al., 2014), data on violence or sexual aggression (Swartout et al., 2015), and numerous studies on the cost of health care, such as costs among patients with depression or anxiety (Halpern et al., 2013; Vasiliadis et al., 2013), costs following brief cognitive behavioral treatment for insomnia (McCrae et al., 2014), and costs of anorexia nervosa (Stuhldreher et al., 2015), among others. Campitelli et al. (2016) also showed how the gamma distribution fits reaction times better than other well-studied distributions.

Although there is a wide variety of probability distributions, the most frequently used distributions involving real data are much fewer in number. The set of exponential distributions is very common in disciplines associated with the health and social sciences. The exponential family includes the normal, exponential, gamma, beta, and lognormal as continuous distributions, and the binomial, multinomial, and negative binomial as discrete distributions. The lognormal distribution, for example, is frequently found in medicine, social sciences, and economics (Limpert et al., 2001).

The normal distribution is the most well-known distribution and the most frequently used in statistical theory and applications. In fact, normality is one of the underlying assumptions of parametric statistical analysis. In practice, however, data can be drawn from other types of distribution, and in order to obtain accurate results researchers have to decide which statistical technique is best suited to the specific distribution of data. Monte Carlo simulation studies are commonly used to identify the robustness of statistical techniques under violation of underlying assumptions. In relation to continuous distributions, numerous simulation studies have analyzed the lognormal distribution (Algina and Keselman, 1998; Keselman et al., 2000; Kowalchuk et al., 2004; Arnau et al., 2012; Oberfeld and Franque, 2013; Bono et al., 2016, among others), and also the exponential distribution (Lix et al., 2003; Arnau et al., 2012). Among discrete distributions, simulation studies have been conducted with binomial (Wu and Wu, 2007; Fang and Louchin, 2013) and multinomial distributions (Kuo-Chin, 2010; Bauer and Sterba, 2011; Jiang and Oleson, 2011). If the results of simulation studies are to be truly useful they need to include the distributions most commonly used in empirical contexts. However, there are very few studies detailing which distributions are most likely to represent data in different disciplines.

The aim of the present study was to determine the frequency of appearance of the most common non-normal distributions used in the health, educational, and social sciences. To this end, we conducted a systematic review of papers published between 2010 and 2015, coding two variables: shape of the distribution and field of study.

## Methods

### Selection of Studies for Inclusion in the Review

The search was carried out in the Web of Science (WOS) database and used the following terms: ‘*nonnormal distribution*’ OR ‘*non-normal distribution*’ OR ‘*nonnormal data*’ OR ‘*non-normal data*’ OR ‘*ordinal data*’ OR ‘*categorical data*’ OR ‘*multinomial data*’ OR ‘*binary data*’ OR ‘*binomial data*’ OR ‘*gamma distribution*’ OR ‘*beta distribution*’ OR ‘*lognormal distribution*’ OR ‘*log-normal distribution*’ OR ‘*log normal distribution*’ OR ‘*exponential distribution*’ OR ‘*binary distribution*’ OR ‘*binomial distribution*’ OR ‘*multinomial distribution*’ OR ‘*nonnormal distributions*’ OR ‘*non-normal distributions*’ OR ‘*gamma distributions*’ OR ‘*beta distributions*’ OR ‘*lognormal distributions*’ OR ‘*log-normal distributions*’ OR ‘*log normal distributions*’ OR ‘*exponential distributions*’ OR ‘*binary distributions*’ OR *‘binomial distributions*’ OR ‘*multinomial distributions.*’ The use of these terms was agreed by two reviewers (first and third author), such that the search strategy employed general descriptors of non-normal distributions, descriptors for ordinal or categorical data, and specific descriptors of the most common non-normal distributions. The term ‘*negative binomial distribution*’ was not included as it was encapsulated by the term ‘*binomial distribution.*’ No restriction on the language of publication was made. The terms included were refined to the following WOS research areas: *Psychology*, *Health Care Sciences Services*, *Education and Educational Research*, *Social Sciences Other Topics*, *Psychiatry, Social Issues*, *Behavioral Sciences*, and *Biomedical Social Sciences*.

The selection of studies, based on title and abstract, was performed independently by two reviewers (first and second author). The following kinds of study were excluded from the review: theoretical studies of a statistical test, new procedures, mathematical development, comparison of models, simulation studies, tutorials, reviews of other authors’ work, comments on other articles, systematic reviews, meta-analyses, studies about the teaching/learning of distributions, software, and studies carried out in areas other than health, education, or social sciences. We also excluded conference abstracts and proceedings, and book reviews. Any articles that did not specify the type of distribution or which referred to the normal distribution were likewise excluded. The inter-rater reliability for selection of articles was assessed with Cohen’s kappa (Cohen, 1968). The weighted kappa was 0.84, which can be interpreted as almost perfect agreement (Landis and Koch, 1977). Disagreements were resolved by discussion.

### Data Extraction

Information about the type of distribution and the field of study was extracted from the content of the abstract and title of the included articles. In the event that more than one distribution was mentioned in an abstract, they were all recorded. Data were extracted independently by two reviewers (first and second author). The inter-rater reliability regarding the type of distribution was 96.5%. Discrepancies were resolved by consensus after reviewing again the abstracts in question; in the event that a consensus could not be reached, the final decision was taken by a third reviewer (fourth author).

## Results

**FIGURE 1:**
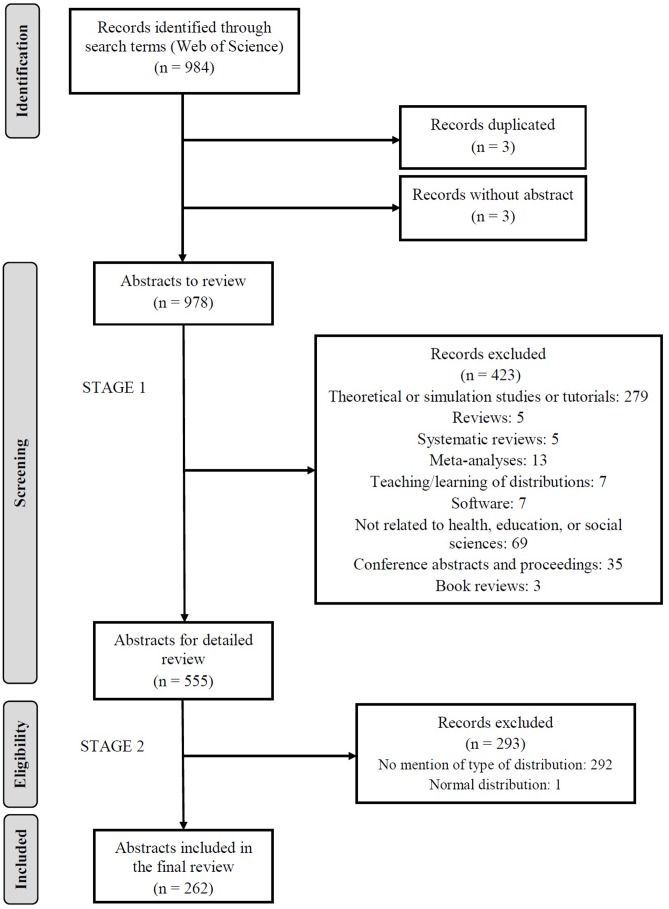
Flow chart of the study selection process.

Of the 984 articles that were initially retrieved we eliminated, in stage 1, three duplicate records, three articles from journals without abstracts, and 423 articles according to the abovementioned exclusion criteria (see Selection of Studies for Inclusion in the Review). In stage 2 we eliminated a further 292 abstracts that made no mention of the type of distribution and one which referred to a normal distribution. **Figure 1** summarizes the numbers of records identified and the reasons for exclusion at each stage. It can be seen that of the 984 records retrieved from the WOS, 262 were included in the review (148 from the area of health, 18 from education, and 96 from the social sciences). Seventeen abstracts referred to two distributions, all of which were counted, and therefore a total of 279 distributions were considered.

**FIGURE 2:**
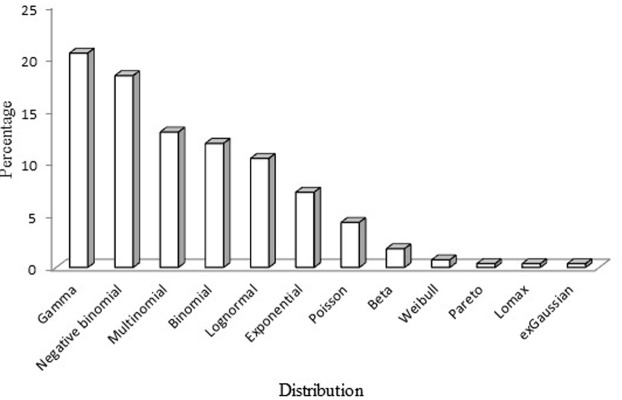
Percentage of the different distributions across the articles retrieved from the Web of Science (WOS) database for the period 2010–2015.

Across the reviewed studies the most common distributions were gamma (*n* = 57), negative binomial (*n* = 51), multinomial (*n* = 36), binomial (*n* = 33), lognormal (*n* = 29), and exponential (*n* = 20). The beta distribution fitted to very few data sets (*n* = 5). Other distributions identified but which had not been considered as search terms were the Poisson (*n* = 12), Weibull (*n* = 2), Pareto (*n* = 1), Lomax (*n* = 1), and exGaussian (*n* = 1). In addition to these distributions, 31 abstracts only indicated that the distribution was non-normal. **Figure 2** shows the percentage of the different types of distribution across the articles included in the review.

## Discussion

The aim of this systematic review of papers published between 2010 and 2015 was to determine the frequency of appearance of the most common non-normal distributions used in the health, educational, and social sciences. The results show that the most frequent distributions are the gamma and the negative binomial, followed by the multinomial, the binomial, the lognormal, and the exponential. The multinomial and binomial distributions show a good fit to data derived from discrete measurement scales, whereas the gamma and negative binomial distributions fit to variables related to health costs or income in social research. These findings extend those obtained by Micceri (1989) and Blanca et al. (2013), who analyzed the distributional characteristics of real data and noted that non-normal distributions are commonly found when working with psychological variables and psychometric measures. Knowing which distributions are most common is important because the type of distribution is a key aspect to consider when choosing an analytical technique.

When researchers know that the distribution which fits their data is non-normal, they should consider using alternatives to classical procedures. One way of modeling the response variable in order to find the type of distribution that best represents the data is to apply what are known as generalized additive models for location, scale, and shape (GAMLSS; Rigby and Stasinopoulos, 2005). This method provides the foundations for further analyses (Campitelli et al., 2016). Other data analysis procedures include robust statistical methods (Wilcox, 2012), generalized linear models (McCullagh and Nelder, 1989) and their extension to mixed models (Stroup, 2013), and linear quantile mixed models (Geraci and Bottai, 2014).

As regards the limitations of this study the search was limited to a specific set of distributions, those considered to be the most common, and it is possible that the type of distributions identified by the review was biased somewhat by the search terms used. However, with the descriptors used we located the most well-known distributions from the exponential family. In order to access the full range of distributions, including the less common ones, we would have had to have applied the search term ‘*distribution*,’ which would have yielded many more types of distribution with a low or very low percentage across studies. One distribution of the exponential family that is of interest but which is not analyzed in our study is the Poisson distribution. This distribution was not included in the systematic review because it is more directly associated with count data and, in such cases, the negative binomial distribution can be used as an alternative (Faddy and Smith, 2011; Smith and Faddy, 2016).

Another limitation is that it is difficult to know whether the data are actually distributed as identified in the title and/or abstract. That is, researchers may have simply assumed particular non-normal distributions based on histograms or frequency distributions, or on a prior decision to apply a particular statistical technique or software. Empirical studies do not always indicate the distribution shape, or the procedure used to identify which distribution fits the data, and neither is a rationale usually given for why a particular non-normal distribution was used. Ideally, studies would report this kind of information so that other researchers from the same applied field have clear knowledge about the distributional properties of the variables under study.

Finally, and as noted in the introduction, the known distributions most widely used in simulation studies are the lognormal and the exponential, although discrete distributions such as the binomial and the multinomial have also been analyzed. In light of the results of this systematic review, future simulation studies examining the robustness and power of different statistical tests should also use the gamma and negative binomial distributions, the two most common forms according to our review. This is important because simulation studies need to include the distributions used in real-world data. Thus, we suggest that researchers who conduct Monte Carlo studies should generate data according to the distributions that are most relevant to the empirical reality of different disciplines.

## Author Contributions

RB was responsible for planning and executing the research activity and for drafting the manuscript, was involved in selecting the search terms to be used in the systematic review, acted as the second reviewer of the systematic review, and wrote the final version of the manuscript. MB was the first reviewer of the systematic review and offered a review of the manuscript’s content. JA was involved in selecting the search terms to be used in the systematic review and supervised the drafting of the manuscript. JG-B supervised the methods of systematic review and the final version of the manuscript, and acted as the third reviewer in the event that the first two reviewers could not reach an agreement regarding the type of distribution. All authors agree to be accountable for the content of the work, and have approved the final version to be published.

## Conflict of Interest Statement

The authors declare that the research was conducted in the absence of any commercial or financial relationships that could be construed as a potential conflict of interest.
